# IEM explained

**DOI:** 10.1080/09515089.2024.2359493

**Published:** 2024-05-31

**Authors:** François Recanati

**Affiliations:** Chaire Philosophie du langage et de l’esprit, Collège de France, Paris, France

**Keywords:** Immunity to error through misidentification (IEM), de se, experience, mode, first person, indexical concepts

## Abstract

In this paper I compare my account of IEM to another one, the Simple View, according to which a judgment is IEM just in case its grounds do not include an identity. The Simple View does not say *why* no identity assumption is needed to ground the singular judgment in the IEM cases; my account is meant to complement it by providing an answer to that question. According to my account, the judgments that are IEM are based on a certain experience, and what they are about is pre-determined by the *mode* of that experience, by the type of experience it is, in a way that leaves no room for an alternative.

## 1.

Consider a judgment in which a property *F* is ascribed to an entity *e*, e.g., “the cat is on the mat”, where *e* is the cat and *F* is the property of being located on the mat. Such a judgment can, in principle, suffer from two kinds of mistakes, one concerning the subject, and one concerning the predicate. The judgment may incorrectly identify the property which the entity *e* actually instantiates; for example the cat may be in the kitchen rather than on the mat. In this case the judgment misidentifies the location of the cat, and we can talk of predicate-misidentification. Or the subject may incorrectly identify the entity which instantiates the property *F*; for example, it may be the dog, rather than the cat, that is on the mat. In this case the judgment misidentifies the animal currently on the mat, and we can talk of subject-misidentification. Of course these mistakes are not exclusive of each other. The person making the judgment may misidentify the animal currently on the mat (seeing the dog, but mistaking it for the cat) and, as a result, misidentify the location of the cat (taking it to be on the mat).

In certain cases, however, *no subject-misidentification seems to be possible*. Such cases exhibit what has been called “immunity to error through [subject-]misidentification” (IEM, for short). For example, imagine a tourist in a hotel room who opens the window and says, with delight, “I see the Eiffel Tower”. The tourist’s judgment may very well be mistaken. She may be hallucinating (rather than seeing) the Eiffel Tower; or she may be seeing some other monument and mistaking it for it. But one particular kind of mistake seems to be ruled out in such a situation: the tourist cannot misidentify *the person seeing* the Eiffel Tower. She cannot wrongly ascribe the visual experience to herself – that sort of mistake simply cannot happen. In such a situation it makes no sense for the tourist to wonder: “Someone is seeing the Eiffel Tower, but is it me?”

Regarding the phenomenon of IEM, introduced into the philosophical literature by Wittgenstein (1958), Strawson (1966) and especially Shoemaker (1968), a number of questions arise. First, what is the extent of the phenomenon? Self-ascriptions of conscious experiences seem to be IEM, but as Evans (1982) pointed out in his discussion of Shoemaker, self-ascriptions of bodily conditions can be IEM too. If, on the basis of proprioception, I judge that my legs are crossed, or that I am standing, it makes no sense for me to wonder: “Someone’s legs are crossed (or: someone is standing), but is it I?” (Evans, 1982, p. 216.) Nor is IEM restricted to *self*-ascriptions. Third-person judgments can be IEM, no less than first-person judgments. If, looking at a certain man who is running like hell, I judge “That man is in a serious hurry”, it makes no sense for me to wonder: “Someone is in a serious hurry, but is it that man?”

The diversity of cases in which IEM is displayed invites a second question or set of questions: what exactly *is* the phenomenon of IEM? Where does it come from? Various answers have been suggested in the literature, including one I myself have put forward (Recanati, 2007b, 2012).^1^ In this paper I will compare my account to another one which several authors have presented as simpler and superior (García-Carpintero, 2013; Morgan, 2012; Wright, 2012). Before I start, however, I need to mention what is perhaps the most fundamental observation regarding the phenomenon of IEM.

Whether or not a given judgment is IEM is not an absolute matter but depends upon the *grounds* on which the judgment is made (Evans, 1982, p. 219).^2^ The judgment “That man is in a serious hurry” is IEM when made directly on the basis of one’s observation of the man’s behavior; the tourist’s judgment “I see the Eiffel Tower” is IEM when made directly on the basis of her visual experience as of the Eiffel Tower; and the judgment “My legs are crossed” is IEM when it is made on the basis of proprioceptive information possessed by the subject “from inside”. These judgments, however, will not be IEM if made on other grounds. For example, if the subject in Evans’ example judges that her legs are crossed on the basis of seeing her legs (or what she takes to be her legs) in the mirror, the judgment will not be IEM. In such circumstances the subject may misidentify the legs she sees as her legs even though they are actually her neighbor’s; so it would make sense for her to wonder: “Someone’s legs are crossed, but is it I whose legs are crossed?”

I now turn to possible theories of IEM, starting with the simplest and most minimal theory. Wright (2012) calls it the “Simple Account” and Morgan (2012) “the Simple Explanation”. Following the editors of this issue I will call it the Simple View.

## 2.

According to the Simple View, a judgment which is susceptible to subject-misidentification (i.e., a judgment which is *not* IEM) is one that is *identification-dependent* (Evans, 1982, p. 180): its grounds include an identity, [*a* = *b*], and that identity may turn out to be false. By contrast, a judgment is IEM just in case its grounds do *not* include an identity.^3^ For example, the contrast is between:

*(not-IEM)*

These legs are crossed (based on visual inspection of the mirror)

These legs = my legsThus, my legs are crossed (based on visual information + the identity assumption)

and

*(IEM)*

My legs are crossed (directly based on proprioceptive information accessible “from inside”, without the help of an identity assumption)

I think the minimal theory is correct as far as it goes, but it does not go very far, or at least, not far enough. It does not say *why* no identity assumption is needed to ground the singular judgment in the IEM cases. My account of IEM is meant to complement the Simple View by providing an answer to the *why* question. I made that point explicit in my response to García-Carpintero (2013):

García-Carpintero contrasts [my] account with the so-called “Simple Account” (…). He says that I offer my account “instead of” the Simple Account, and adjudicates in favor of the latter. But these accounts do not compete. My account explains *why* (…) there is no need for an identity premise in the grounds. (Recanati, 2013, p. 236)

Note that, in a later piece, García-Carpintero granted me that point and acknowledged “the need to explain *in virtue of what* some beliefs can dispense with an identity-judgment in their background” (García-Carpintero, 2018, p. 3315).^4^

Why is it the case, then, that e.g., the first-person judgment “my legs are crossed” can be directly based upon the proprioceptive experience without the help of an identity premise? One possible answer is that, in the IEM cases, the judgment is reached *non-inferentially*. As Crispin Wright puts it,

A genuine singular judgment, [*a* is *F*], can enjoy immunity to error through misidentification (…) if its grounds do not include any *identification* (…). And one circumstance that ensures that, one might suggest, is simply if the judgment is non inferential! (Wright, 2012, p. 254)

Admittedly, there is a sense – indeed, several senses – in which the justification of the judgment “My legs are crossed” is non-inferential when it is directly based on the proprioceptive experience. Likewise for the judgment “I see the Eiffel Tower” when it is based on the visual experience. With respect to the demonstrative judgment “That man is in a serious hurry”, the situation is a little more complex, for the judgment is partly inferential: it is based on the visual experience of the man running like hell, but also depends on a background assumption (that someone who runs like this must be in a serious hurry). So an inference is involved, but not one that exploits an identity premise. In the non-IEM cases, by contrast, the judgment results from an inference from a set of premises that includes an identity assumption. So the Simple View goes.

But that explanation of the difference between the two types of cases is not satisfactory as it stands. We have to say more about what it means to claim that (the justification of) a judgment is, or is not, inferential. As García-Carpintero pointed out, we need to distinguish between what he calls “conscious, explicit, occurrent” (CEO) inferences and merely *tacit* inferences (García-Carpintero, 2001, p. 120); for non-IEM status a tacit inference is enough – no CEO-inference is required.^5^ Not any sort of tacit inference will do, though. Among tacit inferences, García-Carpintero (2001) distinguishes between two classes: personal and sub-personal. A tacit inference of the personal sort is one that satisfies what I dubbed the “availability condition” (Recanati, 2004, pp. 40–44, 162–65): *the subject herself* must be disposed to draw a distinction between the two ingredients which are said to inferentially combine. That is the notion of inference that can be used to single out the non-IEM cases. In typical non-IEM cases, the subject who makes a singular judgment “*e* is *F*” is disposed to retreat to the weaker existential judgment “Something/someone is *F*” if the singular judgment proves untenable.^6^ The subject is disposed to dissociate the existential judgment from the singular judgment, as if the singular judgment rested on the existential judgment plus an extra assumption. That *dissociative disposition* may be in place even though the singular judgment which the subject actually makes does not itself result from a CEO-inference but is based directly upon a perceptual experience (which makes it “non-inferential” in an intuitive but irrelevant sense).

Consider the example I started from – “The cat is on the mat”. We saw that this judgment is susceptible to subject-misidentification (the subject may have misidentified the animal on the mat), yet it is based directly upon a visual experience which the subject takes at face value. No CEO-inference is involved: the subject sees (or takes herself to see) the cat on the mat. Still, her perceptual identification of the cat may be illusory – it may actually be the dog that she is seeing. If challenged, the subject will be disposed to retreat to the weaker judgment *Some animal is on the mat – I thought it was the cat*. In other words, the subject herself is disposed to dissociate the existential judgment “Some animal is on the mat” from the singular judgment “The cat is on the mat”, in such a way that the former may survive if the latter is defeated.

What characterizes the IEM cases is the lack of that dissociative disposition which makes it possible for the existential judgment to survive the singular judgment. As Evans repeatedly puts it in *The Varieties of Reference*, when a judgment is IEM, there is, for the subject herself, “no gap” between the existential judgment and the singular judgment, and no possibility that the former survives if the latter is defeated.^7^ *That* is the phenomenon that has to be explained. Now it *cannot* be explained by invoking the idea that an IEM judgment is non-inferential, for that idea can be cashed out in two ways, as we have just seen, and neither gives us what we need. The standard sense of “non-inferential” (i.e., the fact that the judgment is directly based on an experience and does not involve a CEO-inference) is irrelevant: a judgment may be non-inferential in that sense yet susceptible to subject-misidentification. The other sense of “non-inferential” (the lack of the dissociative disposition) is relevant, but the lack of the dissociative disposition is nothing but the no-gap phenomenon we are supposed to explain; so it cannot be used to provide a non-circular explanation of that phenomenon, which it simply redescribes.^8^

It is true that proponents of the Simple View tend to take a deflationary stance: they doubt that a theory of IEM *as such* has to answer the *why* question I have kept raising. Thus Morgan asks, in a skeptical tone:

What is it about immunity to error through misidentification that makes it look as though it is crying out for some non-obvious explanation? Very often, one’s sense of what *needs* to be further explained, and what can be taken for granted, will partly depend on one’s sense of what it might be *possible* to further explain. Is there something about [IEM] that makes it look as though it might be possible to provide a deeper explanation of it than the Simple Explanation does? (Morgan, 2012, p. 123)

I will provide a positive answer to Morgan’s question in the next section. As for Wright, he thinks that, depending on the sort of judgment at stake, the explanation for why it is IEM may be different from case to case. What constitutes IEM, construed as a *common* feature of these judgments, is simply the no-gap phenomenon, according to Wright; it is not anything more specific that might account for why a particular judgment (e.g., a first person judgment) is IEM. Now I agree with Wright that a certain diversity has to be acknowledged in the IEM ballpark^9^; but I want to maintain that, beyond that diversity, the IEM phenomenon, singled out via a handful of paradigmatic examples, corresponds to a *natural kind*, which calls for a positive and unified explanation – the sort of explanation which Morgan doubts it is possible to provide. To that explanation I now turn.

## 3.

Wright ascribes to Gareth Evans the view that “Immunity to error through misidentification is (…) a feature of all *basic* singular-thought – singular thought that is grounded non-inferentially in observation or other forms of experience” (Wright, 2012, p. 254). Indeed, as we have seen, the paradigmatic examples through which the phenomenon of IEM is generally introduced are instances of judgments directly based upon an experience.^10^ This feature is not sufficient for IEM since, as we have also seen, a judgment directly based on an experience may be susceptible to subject-misidentification (that is the case for the observational judgment that the cat is on the mat). Still, being directly based on experience is an important feature of IEM judgments, and we can make it our starting point.

What is an experience? I will not attempt to answer that difficult question, but will content myself with a brief overview. Experiences, I take it, possess three fundamental features. (A fourth one, their “reflexive” character, will be introduced in the next section.)

First, experiential states are *informational states* in Evans’ terminology – they belong to what he calls the “informational system”:

When a person perceives something, he receives (or, better, gathers) information about the world. By communicating, he may transmit this information to others. And any piece of information in his possession at a given time may be retained by him until some later time. People are, in short and among other things, gatherers, transmitters and storers of information. These platitudes locate perception, communication and memory in a system — the informational system — which constitutes the substratum of our cognitive lives. (Evans, 1982, p. 122)

Informational states (such as perceptual states or memory states – I leave testimony aside in this discussion) represent the world as being thus and so: they have representational content in that they are “imbued with (apparent) objective significance, and with a necessary, though resistible, propensity to influence our actions” (Evans, 1982, p. 123). Furthermore they display a “fundamental (even defining) property” which Evans calls their *belief-independence* (Evans, 1982, p. 123):

The subject’s *being* in an informational state is independent of whether or not he believes that the state is veridical. It is a well-known fact about perceptual illusions that it will continue to appear to us as though, say, one line is longer than the other (in the Muller-Lyer illusion) even when we are quite sure that it is not. Similarly, it may seem to us as though such-and-such an episode took place in the past, even though we now believe our apparent experience of it to have been hallucinatory. (Evans, 1982, p. 123)

Relatedly, it is not up to the subject which informational state he is in. What we perceive or remember systematically (though fallibly) depends upon present or past states of the environment to which the informational state is responsive.^11^ When all goes well, the content of our experience “is directly causally dependent on certain properties of [the objects the experience is of] in such a fashion that one is thereby enabled (…) to form a fairly reliable judgment as to what those properties are” (Lowe, 1996, pp. 102–103). In other words, the informational mechanism is such that “the properties that figure in the content of its output are (to a degree determined by the accuracy of the mechanism) the properties possessed by the objects that are the input to it” (Evans, 1982, p. 125).

Second, experiential states constitutively possess a “sensory” dimension. Experiences, Lowe writes,

involve a sensational or sensuous element. (…) Whether there are states of mind that are *purely* intentional — that is, which are “object-directed” but lack any characteristic sensational element — is debatable; but if there are they are not, I believe, *experiential* states. One possible candidate for the status of a purely intentional state would be knowledge “of” somebody or something. But such a state is obviously not experiential. Someone who *knows* Paris need not on that account be undergoing any particular sort of experience, in the way that someone who *sees* Paris must be. However, connected with this is the fact that the verb “to know”, unlike the verb “to see”, does not admit the present continuous tense - one can be *seeing* Paris, but not *knowing* it. Experiences are *occurrent*, as opposed to dispositional, states of mind (...). But what about occurrent intentional mental states like *thinking of*, or *remembering* somebody or something? These are experiential but surely do not involve *sensation*; so are they not “purely” intentional experiential states? No, because although they do not involve sensation directly, I believe that they do involve it derivatively inasmuch as they involve *imagination*. (Lowe, 1996, p. 97)

According to Lowe, it is the sensuous or quasi-sensuous properties of experience (its phenomenal or qualitative character) that depend systematically upon, indeed covary with, properties of the object the experience is of, thereby enabling the subject to make reliable judgments concerning these properties (Lowe, 1996, pp. 107–108).^12^

Third, experiential states come in a variety of types: there are, for example, perceptual states and memory states, with a distinctive phenomenology. The type of an experiential state is what (following Searle, 1983) I call its *mode* (Recanati, 2007a, 2007b). The mode of an experiential state, to which the subject has transparent access through its phenomenology, is related to its functional role. Thus the function of a perceptual state is to inform the subject about its surroundings, while a memory state carries information about the past state of the subject’s environment. A further distinction pertaining to the mode corresponds to the specific sense modality involved (either in perception or memory): there is, for example, a difference between a visual experience and an auditory or olfactive experience. These modalities are all “exteroceptive”, but there are also internal senses such as those involved in interoception, proprioception, kinesthesis, and introspection. The role of these internal sense modalities (and of the “internal mode” of which they are specifications) is to provide the subject with information about herself. (Importantly, we must also make room for composite modes, since our experiences are typically multi-modal.)

At this point I think we are already in a position to explain the IEM of certain judgments, namely first-person judgments based on experiences in the so-called internal mode. The role of an experiential state in that mode is to provide the subject with information *about herself*. For that reason, a proprioceptive state such as the experiential state of the subject who feels, from inside, that her legs are crossed, can only convey information (or misinformation) about herself: it cannot provide information (or misinformation) about the position of the limbs of *another subject*. Even if we assume that an evil surgeon has connected the subject’s brain to another subject’s body in such a way that the proprioceptive states of the first subject actually track the bodily condition of the second subject, it will still seem to the experiencing subject that *her* legs are crossed, because the (illusory) state she is in is a proprioceptive state and the role of such a state is to inform her of her own bodily condition. Likewise for the other senses which qualify as “internal senses”. The information the state delivers may be illusory (as in the cross-wiring case I have just evoked), but it cannot be (taken to be) information about the condition of another person’s body. As Evans puts it,

we cannot think of the kinaesthetic and proprioceptive system as gaining *knowledge* of truths about the condition of a body which leaves the question of the identity of the body open. If the subject does not know that *he* has his legs bent (say) on this basis … , then he does not know *anything* on that basis. (To judge that someone has his legs bent would be a wild shot in the dark.) (Evans 1982, p. 221)

Admittedly, there is an exception, as Evans acknowledges: the subject may have been *told* about, or may have discovered, the cross-wiring situation she is in, and may have learnt to infer from *her* being in that proprioceptive state that *the other subject*’s legs are crossed. In such a case, however, the subject’s judgment (to the effect that the other subject’s legs are crossed) would *not* be directly based on the proprioceptive experience, taken at face value: it would be reached via an inference and would require the subject to “reflect on the process of information-transmission” (Evans, 1982, p. 128).^13^ Perhaps that abnormal situation could become the new normality, as in the case of a subject who, after some time, gets used to wearing inverted lenses; but that hypothesis takes us far outside the normal functioning of the informational system. What matters for our purposes is that, *within* the proper bounds of the informational system, a proprioceptive state, when taken at face value, can only carry information (or misinformation) about the subject undergoing the state. That means that no identification of the person the information (or misinformation) is about is required. The person in question is, as it were, pre-identified, being determined by the mode of the experiential state.

In his defense of the Simple View Daniel Morgan writes:

What the Simple Explanation most obviously takes for granted is the fact that some judgments are not based on any identification. So, in deciding whether [a purported explanation of IEM] goes beyond the Simple Explanation, the question we need to ask is this: does the explanation *explain* the fact that the judgment in question is not based on any identification? (Morgan, 2012, p. 114)

I think it is pretty clear that we *can* explain that fact by appealing to the idea that the mode of a proprioceptive state already determines which person the state is about, in such a way that *no further identification of that person is required*. There is no gap between the existential judgment, “someone’s legs are crossed”, and the singular judgment “my legs are crossed” because, *when the information that someone’s legs are crossed is gained in the internal mode*, that information can only be about the subject of experience.^14^ By contrast, if the information that someone’s legs are crossed is gained in the visual mode, the question remains open, which person seen in the mirror is such that his or her legs are crossed. It may be the subject of experience, but it may also be some other person, for the good reason that, in a mirror, we may see ourselves but we may also see other persons (and we may mistake one for the other).

The same explanation may be offered for why the judgment “The cat is on the mat”, based on a visual experience, is not IEM: the visual mode does not guarantee that the animal whose location on the mat is visually detected is the cat, the dog, or whatever: an actual identification of the animal is required, so there *is* a gap between the existential judgment “some animal is on the mat” and the singular judgment “The cat is on the mat” when these judgments are based on a visual experience.

## 4.

I have just explained why first-person judgments based on experiences in the internal mode are IEM. Such judgments are immune to subject-misidentification because they don’t involve an identification of their subject; and they don’t involve an identification of their subject because they rest on an experience in the internal mode, i.e., an experience such that the individual whose properties are detected does not have to be identified (since its identity is pre-determined by the mode of the experience). My task is now to show that that explanation of IEM in terms of the mode of the experience suitably generalizes.

Let us start with first person judgments that are IEM even though, contrary to the proprioceptive example discussed in the previous section, they are not based on an experience in the internal mode but on an experience whose mode is exteroceptive. I mentioned the case of the tourist who enjoys a visual experience as of the Eiffel Tower and, based on that experience, judges: “I see the Eiffel Tower”. That judgment is IEM since the subject cannot misidentify *the person seeing* the Eiffel Tower. But how can a judgment based on a visual experience be IEM if the explanation of IEM I have given is correct? According to that explanation, a judgment such as “my legs are crossed” is IEM, when based on a proprioceptive experience, because the mode of that experience is such that the content of the experience can only be about the subject of experience. A visual experience such as that which the subject enjoys when she sees her legs in the mirror works differently: we may see ourselves in the mirror but we may also see other persons (and we may mistake one for the other). The visual mode is exteroceptive; it is not an “internal” mode. So how can the account of IEM I have provided extend to first person judgments based on visual experiences?

My account of IEM extends to such cases because there is a sense in which a visual experience too is bound to be about the subject of experience. To be sure, *what the subject sees* (the *content* of her visual experience) need not involve the subject herself: she may see some other person in the mirror, or the Eiffel Tower. That is why a first-person judgment based on a visual experience may suffer from subject-misidentification. But what the subject sees, *whether or not it involves the subject herself*, is bound to be *something present in the subject’s environment and visible to her* – otherwise she wouldn’t see it. In that looser sense, the content of the subject’s visual experience is bound to be “about herself”: it is about *her* environment. As John Perry wrote in “Perception, Action, and the Structure of Believing” (Perry, 1986a), “The information that we get at a certain spot in the world is information about objects in the neighborhood of that spot in a form suitable for the person in that spot” (Perry, 1993, pp. 148–9).^15^ For that reason, a first-person judgment based on a visual experience is IEM when the subject self-ascribes the property of seeing whatever she takes herself to see, e.g., the Eiffel Tower. The subject may not actually see (but merely hallucinate) the Eiffel Tower, or it may not be the Eiffel Tower but some other monument that she sees; but she can only see what is visible to *her* and affects *her* visual apparatus. The identity of the seer is not up for grabs. (Of course, we can imagine a fantastic situation in which an evil surgeon has connected the subject’s brain to another subject’s visual apparatus, but such a scenario takes us outside the normal functioning of perception.)

The same analysis applies to first-person judgments based on memory. If I remember having visited Rome with Julia, I may be mistaken in many ways. It may be an apparent but illusory memory (such a visit was planned but never occurred); or it may be a memory of a visit to Athens, not Rome; or it was not Julia, but Mary. But what cannot happen is that it wasn’t me, but someone else. Based on the memory experience, I cannot wonder: “someone visited Rome with Julia, but was it me?” Again, the explanation is simple. The function of a memory state is to retain information about episodes the remembering subject experienced in the past. The identity of the subject whose past is at issue is not up for grabs: it is the remembering subject who remembers episodes *she* experienced in the past. That’s how memory works. We can imagine a fantastic situation in which a subject “quasi-remembers” episodes in the life of another subject (Shoemaker, 1970), but that scenario takes us outside the normal functioning of memory.

Perception and memory both are “reflexive states” (Recanati, 2007b, pp. 161–66), in that the content of the state has to be evaluated with respect to the subject of the state (the person *in* the state).^16^ Thus the content of a memory is to be evaluated as veridical or not by considering whether, in the past experience of *the remembering subject*, there is an episode corresponding to the content of the memory. Likewise, the content of a perceptual state is to be evaluated as veridical or not by comparing it to *the perceiver’s* actual environment, and more specifically, to that part of the perceiver’s environment which causally affects her sensory apparatus. If the subject has a perceptual experience as of a state of affairs A, the fact that A obtains *somewhere* (e.g., in Tibet, or on a distant planet) does not suffice to make the perceptual experience veridical: it has to obtain in the subject’s vicinity and causally affect the subject in the right way (Searle, 1983). In general, a reflexive state can’t be veridical unless there is a certain relation *R* (dictated by the mode of the state) between *the subject of experience* and the state of affairs that is the content of experience. In veridical visual perception the subject *sees* the state of affairs; in veridical visual memory the subject *has seen it* in the past. The subject in a perceptual or memory state is automatically (though defeasibly) disposed to self-ascribe that relation *R*, dictated by the mode, to the content of the state. Such a self-ascription may be mistaken or illusory, if the relevant relation does not actually obtain, yet it is IEM: the subject may be mistaken in judging e.g., that she sees the Eiffel Tower, but the mistake can’t consist in a misidentification of the person who sees. The identity of the person who stands in the relation *R* to the content of the state is pre-determined by the nature of the state qua reflexive state.^17^

## 5.

All the cases we have dealt with thus far are cases of first-person judgment, and many theorists take IEM to be a feature of first-person thought. But the phenomenon has broader scope: some judgments are IEM even though they are not first-person judgments. I will now show that my account of IEM in terms of mode extends to such cases as well.

Perception is not merely a reflexive state, the content of which concerns the subject of the state. In contrast to memory, the content of which is to be evaluated with respect to a *past* situation, the content of a perceptual experience is to be evaluated with respect to the situation the experiencing subject is in at the time at which the perceptual experience occurs. If, at time *t*, I see (or hear, or feel) that it is raining, a judgment based on that experience – a judgment I could express by saying “it is raining” – is true only if it is raining at *t*. I can make that explicit by means of a temporal adverb: “it is raining now”. When the time of evaluation dictated by the mode of the perceptual experience is thus made explicit in the content of the judgment based on that experience, the judgment is question is IEM with respect to the time in question. The subject may be hallucinating, or mistaking snow for rain, but cannot go wrong in thinking that whatever meteorological event is happening (if anything is happening) is happening now. “Now”-judgments can thus be IEM for the same reason that certain first-person judgments are IEM: because the mode of the experience on which the judgment is based determines that it is about (the subject’s situation at) *the time at which the experience occurs*. The same considerations apply to “here”-judgments, which can be IEM too. So IEM is not a property of (certain) first-person judgments, but a property of (certain) judgments involving *indexical concepts* – the concepts expressed by indexical words such as “I”, “here” and “now”.

The need to bring indexical concepts into the picture invites a refinement of the theory I have presented. I explained IEM by saying that certain things (e.g., the *person* whose bodily condition is detected through proprioception, or the *time* at which a perceived event occurs) do not have to be independently “identified” (and so cannot be “misidentified”) because the mode of the experience pre-determines their identity. The time of a perceived event can only be the time of the perceptual experience, and the person whose bodily condition is proprioceptively detected can only be the subject of experience. But a judgment is IEM only if, in that judgment, the relevant thing is *thought of in the right way*, under the right concept. If, on the basis of a proprioceptive experience which reveals that my legs are crossed, I judge “François Recanati’s legs are crossed”, the judgment is true (since my legs are crossed, and I am François Recanati) but it is not IEM. It is not IEM because I might be wrong to identify myself as François Recanati. Or if, at 3 pm, I have a perceptual experience of rain, and judge “It is raining at 3pm”, my judgment is true but it is not IEM. I might be right that it is currently raining, but wrong to identify the current time as 3pm. For the judgment to be IEM, the relevant thing (the thing that cannot be misidentified) must be thought of under an indexical concept: I must think of myself as myself (as “I”), and I must think of the relevant time as “now”.

Why is there such a connection between IEM and indexical concepts? The connection has been rightly emphasized by those who advocate a “meta-semantic” explanation of IEM in terms of the concepts involved.^18^ In my framework, the connection is explained by pointing out that

[Indexical] concepts are ways of thinking of objects through contextual relations the subject bears to them, and these relations to the objects of thought are implied by the experiential modes. In the [IEM] judgment “It is raining here”, the place is identified indexically via the contextual relation the subject bears to it : the relation of being in that place. Now the subject has to stand in that relation to a place in order to gain information perceptually from it (i.e., in order to know, through perception, what happens in that place). Similarly, in the [IEM judgment] “I am standing”, the object to which the property of standing is ascribed is identified indexically via the relation of identity to the thinking subject ; and the subject can gain information about an object on the proprioceptive/kinaesthetic mode only if he or she stands in that relation (identity) to that object. So, to cut a long story short, the reason why [IEM judgments] have to involve indexical concepts is that indexical concepts are those concepts which exploit the contextual relations to the environment on which experience implicitly depends (Recanati, 2012, pp. 195–96).

What about demonstrative judgments? Why is “That man is running like hell” IEM? A demonstrative concept rests on a certain relation to the referent, namely the attentional relation. To think of an object under a demonstrative concept one has to have one’s attention focused on that object (Campbell, 1997). Now the experience on which the judgment “That man is running like hell” (or, for that matter, “That man must be in a serious hurry”) is based is a perceptual experience involving a focusing of attention on the man in question. We can speak of a perceptual-attentional (PA) experience in that sort of case: it is an experience in which the subject gains information about an object by e.g., looking at it. So in this example too the concept deployed in the judgment (the demonstrative concept “that man”) is a concept whose reference is fixed via the same relation to the object as that which the experience exploits.

When the relevant object is thought of under an indexical concept corresponding to its role in the experience on which the judgment involving the concept is based, no further identification of that object takes place – that is why the judgment is IEM. But further identification may occur, and with it the possibility of misidentification. If, identifying the running man as David Chalmers, the subject judges “David Chalmers is running like hell” or “David Chalmers is in a serious hurry”, the judgment is no longer IEM even though the referent stays the same. It is no longer IEM because, even if the man attended to actually is David Chalmers, he might have been someone else. Were the subject given good evidence that the man is not David Chalmers, the subject would retreat to the weaker, existential judgment: *Someone* is running like hell (and must be in a serious hurry). No such retreat is possible when the man is thought of merely as “that man”, that is, as the man the thinker is currently focusing his attention on. In such a case there is “no gap” between the existential information that someone is running like hell, *gained by looking at the man in question*, and the singular information that that man is running like hell.

## 6.

According to the “distribution” principle (Recanati, 2007b, pp. 34–35, 2008, pp. 42–43), what the *mode* of an experiential state pre-determines need not be represented in the *content* of the state, even though it contributes to the veridicality conditions of the state. Thus my perception of a state of affairs is veridical only if that state of affairs obtains in *my* surroundings, but, assuming I am not part of *what* I see, the content of my experience may be considered as “selfless” even though I am involved as the person who sees the state of affairs in question. Or, as I prefer to say, there are two levels of content: the “complete” content of the experience factors in the contribution of the mode, while the explicit content abstracts from it. This two-level theory is reminiscent of Lewis’s approach to *de se* thoughts (Lewis, 1979). According to Lewis, the content of a *de se* thought is selfless, strictly speaking: it is a *property* which the subject of the state self-ascribes. The “self-ascription” bit is contributed by the assertoric or judicative force of the utterance or thought and it is what makes the complete content of that utterance or thought first-personal: the property that is the explicit content is ascribed to the subject of experience, and the judgment or utterance is correct only if the subject actually has the property. On that pattern, I argued that the (explicit) content of a proprioceptive state (or any experience in the internal mode) is a property or property instance, say a bodily condition like *having one’s legs crossed*. A full subject-predicate proposition is generated only at the level of complete content: the property that is the explicit content of the experience is self-ascribed by the subject of experience in virtue of the internal mode of that experience.^19^

Going further, I proposed to generalize that sort of account and I argued that even demonstrative judgments can be construed as based on an experience the explicit content of which is less than a full subject-predicate proposition.^20^ The relevant experience is a PA-experience: the subject gains perceptual information about an object *by attending to that object*. The explicit content of such an experience is a set of properties or property instances which the subject detects by attending to the object; and, as (Campbell, 1997) emphasizes, it is the attentional act itself that binds the properties to the object it determines (the object the subject is attending to).

That proposal has failed to convince philosophers otherwise sympathetic to my attempt to go beyond the simple account and provide a genuine explanation of IEM. I think their reluctance to accept the extension of my explanation to demonstrative judgments may stem from a confusion (for which I may be partly responsible) between the content of the experience and the content of the *judgment* based on it.^21^ In the demonstrative case the contribution of the mode (the demonstrated object, determined by the attentional act) is made explicit in the judgment, so the content of the demonstrative *judgment* is a full subject-predicate proposition. I am, therefore, *not* claiming that the content of the demonstrative judgment is a property or a set of properties. If that is the source of the reluctance, the reluctance is unjustified.

But there is another potential source of worry which I take much more seriously. It may be doubted that the content of the PA *experience* is a property or set of properties. Admittedly, it is the attentional act which binds the observed properties to their bearer (the attended object), but the bearer in question is nevertheless part of the content of the perceptual experience. That has to be so if the content of the perceptual experience is, as I claim, *what we perceive*. In our demonstrative example, what the subject perceives is *the man* running like mad; so there is good reason to question the claim that the experience only has ‘thetic’ content. (On that notion, see footnote 20.)

But perhaps what we should question is the claim that the content of a perceptual experience is *what we perceive*. Evans (1982) rejects that equation and introduces a distinction between the content of an experience and what the experience is of. For him, the content of a perceptual state is similar to that of a photograph:

[The content of a photograph can be specified] by an open sentence in one or more variables (the number of variables corresponding to the number of objects in the photograph). Thus if we are concerned with a photograph of a red ball on top of a yellow square, then the content of the photograph can be represented by the open sentence

Red (x) & Ball (x) & Yellow (y) & Square (y) & On Top Of (x, y)

(Evans 1982: 125)

Of course, in order to judge the accuracy of the photograph, we have to consider the objects the photograph is *of* and compare the photograph to them. But, Evans insists,

I do not believe that a specification of the content of a photograph should make reference to the object or objects that it is of. A photograph should not be said to represent, e.g., that *a* and *b* are such that the former is *R* to the latter — at least not in the way in which a painting may be said to represent, e.g., that Christ is on the cross. We see, here, the need for a distinction between, on the one hand, an *a*-representation (i.e. a species of particular-representation, in a specification of whose content mention of *a* would figure: something which represents, or misrepresents, *a*), and, on the other, something which, without being an *a*-representation, is a representation *of a*. (Evans, 1982: 125n)

Tyler Burge has criticized Evans’ analogy, on the grounds that “the representational content of perception constitutively has a singular element” (Burge, 2010, p. 381). According to Burge, this follows from the fact that “whether a perceptual state instance is accurate or not depends on whether there are causally relevant particulars and, if there are, whether those particulars are correctly characterized and grouped.” But this is something Evans accepts. Like a photograph, a perceptual state “ is *of* those objects with which we have to compare it in order to judge [its] accuracy” (Evans, 1982, p. 125), but, he points out, we should not presuppose that a specification of the *content* of the state must make reference to those objects. The two-level framework makes it possible to reconcile the two perspectives: the complete content of a perceptual state is constitutively singular, but its explicit content is arguably thetic.

Fortunately, that issue does not have to be settled in the context of our present discussion. Whether or not PA experiences, or perceptual experiences more generally, have thetic content, this has no direct bearing on the explanation of IEM my theory provides, nor on its extension to the demonstrative case. The explanation consists in the following claim: in all cases of IEM, including the demonstrative case, the relevant feature (that which can’t be misidentified, e.g., the person whose bodily condition is proprioceptively detected) is a feature contributed by the *mode* of the experience, by the type of experience it is, *in a way that leaves no room for an alternative*. That is why it does not need to be identified, and cannot be misidentified. Whether or not that feature is represented in the content (of the experience, or of the judgment based on it) is a further issue, which can be bracketed since the explanation of IEM my account provides does not rest on it.^22^

## Notes

1. See also (Recanati, 1993, pp. 88–89; 2009, 2010, 2013, pp. 234–41).

2. Although very widely shared in the current debate on IEM, this assumption seems incompatible with the way Wittgenstein treats this phenomenon. In this paper, however, I am not concerned with Wittgensteinian exegesis.

3. Morgan (2012), p. 105: “The Simple Explanation just consists in the observation that a judgment will be immune to error through *misidentification* when it is not based on an *identification*. The Simple Explanation may not appear very exciting – or perhaps even very explanatory. However what it says does at least seem true.” Wright (2012), p. 260: “According to the Simple Account, IEM is, at root, a phenomenon of basic – non-inferential – singular thought. Errors of misidentification, by contrast, are possible only when (…) the justificational structure of a singular thought (is inferential and) exploits, or warrants, a false identification.”

4. “I want to agree with Recanati that the IEM of *de se* thoughts should be explained on the basis of an account of those thoughts, even if I ultimately disagree with him [about] the required substantive account” (García-Carpintero, 2018, p. 3312). Like Peacocke (2012, 2014), García-Carpintero offers a “meta-semantic” account which, although couched in a different framework, is in many ways similar to mine. Space limits prevent me from comparing my account to theirs, but I will say a word about meta-semantic accounts in section 5.

5. “For a judgment A to be epistemically grounded in B does not require that the subject consciously experience his coming to judge A as a result of an inference in part from B” (García-Carpintero, 2018, p. 3316).

6. I speak of “typical” non-IEM cases because, as an anonymous reviewer pointed out, “we can imagine a non-IEM case where the agent is so strongly convinced that *e* is *F* that – were they told that *e* is not *F* — they would rather believe that they are dreaming rather than retreat to the existential judgment.”

7. See Evans (1982), pp. 180, 182, 221, 245.

8. An anonymous reviewer objects to that line of criticism: “At the outset of the paper, IEM was characterized in terms of the impossibility of a certain kind of error/the senselessness of certain kind of questions or concerns. Isn’t there enough distance between these phenomena, so characterized, and the lack of dissociative dispositions to allow us to say that the explanation is non-circular?” I agree that it is not circular to cash out the senselessness of the question “something is *F*, but is it *e*?” in terms of the no-gap phenomenon (characterized as the lack of the dissociative disposition), but I maintain that the no-gap phenomenon itself cannot be explained, without circularity, by appealing to the non-inferential character of the judgment at stake.

9. I already mentioned the distinction between bodily and mental self-ascriptions, and between self-ascriptions and other-ascriptions, all of which can be IEM. In addition there are well-known distinctions between (immunity to) *de re* misidentification and (immunity to) *wh*-misidentification, and between logical IEM and contingent (de facto) IEM. (For references, see the editors’ first introductory paper: “Immunity to Error through Misidentification I: Nature and Scope”.) My account is intended to cover all these cases.

10. Discussions of IEM generally mention a few paradigmatic examples and fix the reference of the term “IEM” demonstratively via these examples (see e.g., the list of examples in Peacocke, 2012, pp. 145–46). The aim of my account is to provide a unified explanation of these paradigmatic examples and the natural kind they arguably instantiate. However, it may be that other, non-paradigmatic examples in which subject-misidentification also seems impossible don’t belong to the same kind and call for a different type of explanation. (To that extent Wright may be right.) Thus, Walton reminds us, “Wittgenstein observed that when a person imagines King’s College on fire, there may be no room for questioning his claim that it is King’s College which he imagines” (Walton, 1990, p. 29; for discussion see Recanati, 2007b, pp. 196–99, where it is argued that “what one’s imagination is about is a matter of intentional stipulation and does not have to be identified”). Likewise, some authors have argued that certain judgments *not* based on experience, e.g., mathematical judgments about the natural numbers, may be IEM too (see Coliva, 2017; Palmira, 2019; Wright, 2012). Both types of case fall beyond the scope of this paper.

11. On the idea of “responsiveness”, see Lowe (1996), pp. 106–107

12. On the sensory dimension of experience, see Peacocke (1983): ch. 1.

13. Such reflective cases are discussed by Evans in several places (see Evans, 1982, pp. 185, 221, 239, 244–45).

14. “The subject is using ways of finding out about the world that are, as we might say, ‘dedicated’ to the properties of one particular object, namely that very person. They are not ways of finding out that could be equally well applied to any of a range of objects. It is for that reason that although the subject using such a way of finding out can make a mistake, it could not be a mistake about who is in question” (Campbell, 1999, p. 95).

15. In “Thought without Representation”, published the same year (Perry, 1986b), Perry writes: “What each of us gets from perception may be regarded as information concerning ourselves” (Perry, 1993, p. 219).

16. The expression “reflexive state” comes from Higginbotham (1995), but the definition I have just given is mine.

17. Are all experiential states reflexive states? It depends on whether we construe imagination as an experiential state or, as I prefer, merely as the simulation of an experiential state; for imagination does not obey the “reflexive constraint” which governs experiential states such as perception or memory. See Recanati (2007b), pp. 206–7.

18. On meta-semantic accounts, see the editors’ second introductory paper: “Immunity to Error through Misidentification II: Explanation and Significance”.

19. Peacocke gives as example the experience of hunger, which rationally justifies the self-ascription of the property of being hungry (Peacocke, 2012, p. 148). The content of an experience of hunger is, evidently, *hunger*, even though the content of the judgment based on it is *I am hungry*.

20. Such a content I call, following Brentano (1874), *thetic* content. It corresponds to Strawson’s notion of “feature-placing” (Strawson, 1959). According to Brentano and Strawson, the content of e.g., the judgment that it is raining is not a full subject-predicate proposition but something more primitive, which does not involve the identification of any particular of which something is predicated. On the convergence between Brentano and Strawson, see my Collège de France lectures for 2024 (https://www.college-de-france.fr/fr/agenda/cours/la-structure-des-contenus-mentaux).

21. I say I am partly responsible because, in *Perspectival Thought* (Recanati, 2007b), I claimed that the content of an IEM judgment is “thetic” (selfless, in the particular case of *de se* thoughts), thus transferring to it a property of the content of the experience it is based on. That was a mistake and, in later writings (Recanati, 2009, 2010, 2012), I corrected it: I argued that the contribution of the experiential mode can be made explicit in the judgment, via indexical concepts, while preserving IEM. In such a case at least the content of the IEM judgment is a full subject-predicate proposition.

22. Work on this paper has been conducted as part of two projects: the *EssInTex* project, funded by the French *Agence Nationale de la Recherche* (ANR-22-CE93-0004), and the *Mental Files: New Foundations* project, funded by the European Research Council (ERC-2023-AdG FILE_101141267). The paper has benefitted from the insightful remarks of two reviewers, as well as from Manuel García-Carpintero’s comments.

## References

[cit0001] Brentano, F. (1874). *Psychologie vom empirischen Standpunkt*. Duncker & Humblot.

[cit0002] Burge, T. (2010). *Origins of objectivity*. Clarendon Press.

[cit0003] Campbell, J. (1997). Sense, reference and selective attention. *Proceedings of the Aristotelian Society*, 97(1), 55–74. 10.1111/1467-8349.00019

[cit0004] Campbell, J. (1999). Immunity to error through misidentification and the meaning of a referring term. *Philosophical Topics*, 26(1), 89–104. 10.5840/philtopics1999261/237

[cit0005] Coliva, A. (2017). Stopping points: ‘I’, immunity and the real guarantee. *Inquiry*, 60(3), 233–252. 10.1080/0020174X.2017.1261999

[cit0006] Evans, G. (1982). *The varieties of reference*. J. McDowell (Ed.) Clarendon Press.

[cit0007] García-Carpintero, M. (2001). Gricean rational reconstructions and the semantics/pragmatics distinction. *Synthese*, 128(1/2), 93–131. 10.1023/A:1010301706013

[cit0008] García-Carpintero, M. (2013). The self file and immunity to error through misidentification. *Disputatio*, 5(36), 191–206. 10.2478/disp-2013-0017

[cit0009] García-Carpintero, M. (2018). *De Se* Thoughts and immunity to error through misidentification. *Synthese*, 195(8), 3311–3333. 10.1007/s11229-015-0817-y

[cit0010] Higginbotham, J. (1995). Tensed thoughts. *Mind and Language*, 10(3), 226–249. 10.1111/j.1468-0017.1995.tb00012.x

[cit0011] Lewis, D. (1979). Attitudes *De Dicto* and *De Se*. *The Philosophical Review*, 88(4), 513–543. 10.2307/2184843

[cit0012] Lowe, J. (1996). *Subjects of experience*. Cambridge University Press.

[cit0013] Morgan, D. (2012). Immunity to error through misidentification: What does it tell us about the *de se*? In S. Prosser & F. Recanati (Eds.), *Immunity to error through misidentification: New essays* (pp. 104–123). Cambridge University Press.

[cit0014] Palmira, M. (2019). Arithmetic judgements, first-person judgements, and immunity to error through misidentification. *Review of Philosophy and Psychology*, 10(1), 155–172. 10.1007/s13164-018-0395-2

[cit0015] Peacocke, C. (1983). *Sense and content: Experience, thought, and their relations*. Clarendon Press.

[cit0016] Peacocke, C. (2012). Explaining *de se* phenomena. In S. Prosser & F. Recanati (Eds.), *Immunity to error through misidentification: New essays* (pp. 144–157). Cambridge University Press.

[cit0017] Peacocke, C. (2014). *The mirror of the world*. Oxford University Press.

[cit0018] Perry, J. (1986a). Perception, action, and the structure of believing. In R. Grandy & R. Warner (Eds.), *Philosophical grounds of rationality: Intentions, categories, ends* (pp. 333–361). Clarendon Press. Reprinted in Perry (1993) p. 121-49.

[cit0019] Perry, J. (1986b). Thought without representation. *Proceedings of the Aristotelian Society*, 60(1), 137–166. Reprinted in Perry (1993) p. 205-19. 10.1093/aristoteliansupp/60.1.137

[cit0020] Perry, J. (1993). *The problem of the essential indexical and other essays*. Oxford University Press.

[cit0021] Recanati, F. (1993). *Direct reference: From language to thought*. Blackwell.

[cit0022] Recanati, F. (2004). *Literal meaning*. Cambridge University Press.

[cit0023] Recanati, F. (2007a). Content, mode, and self-reference. In S. Tsohatzidis (Ed.), *John Searle’s philosophy of language : Force, meaning, and thought* (pp. 49–63). Cambridge University Press.

[cit0024] Recanati, F. (2007b). *Perspectival thought. A plea for moderate relativism*. Oxford University Press.

[cit0025] Recanati, F. (2008). Moderate relativism. In M. Kölbel & M. Garcia-Carpintero (Eds.), *Relative truth* (pp. 41–62). Oxford University Press.

[cit0026] Recanati, F. (2009). *De re* and *de se*. *Dialectica*, 63(3), 249–269. 10.1111/j.1746-8361.2009.01194.x

[cit0027] Recanati, F. (2010). Le soi implicite. *Revue de Métaphysique et de Morale*, n° 68(4), 475–494. 10.3917/rmm.104.0475

[cit0028] Recanati, F. (2012). Immunity to error through misidentification: What it is and where it comes from. In S. Prosser & F. Recanati (Eds.), *Immunity to error through misidentification: New essays* (pp. 180–201). Cambridge University Press.

[cit0029] Recanati, F. (2013). Mental files: Replies to my critics. *Disputatio*, 5(36), 207–242. 10.2478/disp-2013-0018

[cit0030] Searle, J. (1983). *Intentionality*. Cambridge University Press.

[cit0031] Shoemaker, S. (1968). Self-reference and self-awareness. *The Journal of Philosophy*, 65(19), 555–567. 10.2307/2024121

[cit0032] Shoemaker, S. (1970). Persons and their past. *American Philosophical Quarterly*, 7(4), 269–285.

[cit0033] Strawson, P. F. (1959). *Individuals: An essay in descriptive metaphysics*. Methuen.

[cit0034] Strawson, P. F. (1966). *The bounds of sense*. Methuen.

[cit0035] Walton, K. (1990). *Mimesis and Make-Believe*. Harvard University Press.

[cit0036] Wittgenstein, L. (1958). *The blue and brown books*. Blackwell.

[cit0037] Wright, C. (2012). Reflections on François Recanati’s ‘Immunity to error through misidentification: What it is and where it comes from’. In S. Prosser & F. Recanati (Eds.), *Immunity to error through misidentification: New essays* (pp. 247–280). Cambridge University Press.

